# The oncogenic role and regulatory mechanism of PGK1 in human non-small cell lung cancer

**DOI:** 10.1186/s13062-023-00448-9

**Published:** 2024-01-02

**Authors:** Tian Tian, Yahui Leng, Bingbing Tang, Xiaoxia Dong, Qiulei Ren, Jingyin Liang, Tianhui Liu, Yanni Liu, Wenxiao Feng, Song Liu, Yang Zhou, Hongyan Zhao, Li Shen

**Affiliations:** 1 Department of Biochemistry, School of Basic Medicine, Hubei University of Medicine, Shiyan, 442000 Hubei China; 2 Department of Clinical Oncology, Taihe Hospital, Hubei University of Medicine, Shiyan, 442000 Hubei China

**Keywords:** NSCLC, Oncogene, Malignant progression, PGK1

## Abstract

**Background:**

Phosphoglycerate kinase 1 (PGK1) is a metabolic enzyme that participates in various biological and pathological processes. Dysregulated PGK1 has been observed in numerous malignancies. However, whether and how PGK1 affects non-small cell lung cancer (NSCLC) is not yet fully elucidated.

**Methods:**

Herein, the non-metabolic function of PGK1 in NSCLC was explored by integrating bioinformatics analyses, cellular experiments, and nude mouse xenograft models. The upstream regulators and downstream targets of PGK1 were examined using multiple techniques such as RNA sequencing, a dual-luciferase reporter assay, Co-immunoprecipitation, and Western blotting.

**Results:**

We confirmed that PGK1 was upregulated in NSCLC and this upregulation was associated with poor prognosis. Further in vitro and in vivo experiments demonstrated the promoting effects of PGK1 on NSCLC cell growth and metastasis. Additionally, we discovered that PGK1 interacted with and could be O-GlcNAcylated by OGT. The inhibition of PGK1 O-GlcNAcylation through OGT silencing or mutation at the T255 O-GlcNAcylation site could weaken PGK1-mediated NSCLC cell proliferation, colony formation, migration, and invasion. We also found that a low miR-24-3p level led to an increase in OGT expression. Additionally, PGK1 exerted its oncogenic properties by augmenting ERK phosphorylation and MCM4 expression.

**Conclusions:**

PGK1 acted as a crucial mediator in controlling NSCLC progression. The miR-24-3p/OGT axis was responsible for PGK1 O-GlcNAcylation, and ERK/MCM4 were the downstream effectors of PGK1. It appears that PGK1 might be an attractive therapeutic target for the treatment of NSCLC.

**Supplementary Information:**

The online version contains supplementary material available at 10.1186/s13062-023-00448-9.

## Background

Lung cancer, comprising small cell lung cancer and non-small cell lung cancer (NSCLC), is the most prevalent malignant neoplasm in the respiratory system [1]. The World Health Organization predicts that lung cancer will result in 2.2 million new cases and 1.8 million fatalities by 2020 [2]. Despite the progress made in contemporary treatments, the prognosis for lung cancer is still unfavorable. Merely 10–20% of patients manage to survive beyond a period of five years from the time of their initial diagnosis. A significant proportion of patients succumb to local recurrence or metastasis. Consequently, further research into the molecular mechanisms that cause lung cancer is urgently needed.

Phosphoglycerate kinase 1 (PGK1) is an essential metabolic enzyme that catalyzes the conversion of 1,3-bisphosphoglycerate into 3-phosphoglycerate, thereby generating ATP during glycolysis [3]. The canonical function of PGK1 is to participate in the modulation of glycolysis [4]. PGK1 also regulates angiogenesis, autophagy initiation, DNA repair, the binding of plasminogen, one-carbon metabolism, and serine biosynthesis. The aberrant expression or different post-translational modifications of PGK1 influences various diseases, such as neurological impairment, hereditary non-spherocytic hemolytic anemia, parkinsonism, and myopathy. It has been discovered that PGK1 has oncogenic properties and is amplified in various human cancers, including renal clear cell carcinoma [5], ovarian cancer [6], breast cancer [7], colon cancer [8], and liver cancer [9]. In recent years, the association between PGK1 and lung cancer has garnered increasing attention. For instance, a meta-analysis has demonstrated that PGK1 affects lung adenocarcinoma prognosis [10]. Another study has reported a strong correlation between the upregulation of PGK1 and the migratory potential of lung cancer cells [11]. Nevertheless, the precise role of PGK1 in NSCLC is not yet fully elucidated.

Glycosylation, a ubiquitous post-translational modification of proteins, is estimated to occur in over 50% of all eukaryotic proteins [12]. As a distinctive form of glycosylation, O-GlcNAcylation adds GlcNAc to serine or threonine residues on proteins. The influence of O-GlcNAcylation on protein stability, activity, and subcellular localization has been well-established through a growing body of research. Thus far, a multitude of proteins have been identified as being O-GlcNAcylated [13, 14]. In the context of colon cancer, it has been confirmed that PGK1 may undergo reversible and dynamic modification with O-GlcNAc at threonine 255 (T255) [8]. However, the current understanding of whether O-GlcNAcylation modifies PGK1 in NSCLC is considerably restricted.

Hence, the first objective of this work was to assess the contribution of PGK1 to the malignant progression of NSCLC. The secondary objective was to explore the potential involvement of O-GlcNAcylation in PGK1-mediated NSCLC progression. The tertiary objective was to identify the critical molecules and signaling pathways that are relevant to PGK1. The outcomes of this work furnish new insights into the non-metabolic role and regulatory mechanism of PGK1 in NSCLC.

## Methods

### Bioinformatics and microarray analyses

The present study acquired publicly available sequencing data from reputable sources, including The Cancer Genome Atlas (TCGA), Gene Expression Omnibus (GEO), Genotype-Tissue Expression (GTEx), cBioPortal for Cancer Genomics, Gene Expression Database of Normal and Tumor tissues 2 (GENT2), Clinical Proteomic Tumor Analysis Consortium (CPTAC), Human Protein Atlas (HPA), Cancer Cell Line Encyclopedia (CCLE), LinkedOmics, and Kaplan–Meier Plotter. The study utilized a cDNA microarray (MecDNA-HLugC042) obtained from Outdo Biotech (Shanghai, China).

## Cell culture and transfection

Human NSCLC cells (A549 and H1299) and human embryonic kidney 293 T cells were provided by Procell (Wuhan, China). The culture medium was DMEM (Gibco, Carlsbad, CA, USA) containing 10% FBS. To achieve gene overexpression, the full-length human PGK1, O-GlcNAc transferase (OGT), or Minichromosome maintenance complex component 4 (MCM4) gene was amplified via PCR and subsequently subcloned into a pEnter vector (Vigene Biosciences, Jinan, China). Transfections were carried out in A549 and H1299 cells utilizing jetPRIME reagent (Polyplus, Illkirch, France). To accomplish gene knockdown, shRNA oligos targeting PGK1, OGT, or MCM4 were inserted into the pLKO.1 lentiviral vector (Addgene, Cambridge, MA, USA). Lentiviral particles were generated by transiently transfecting 293 T cells with jetPRIME. Then the packaged lentiviruses were harvested and utilized to infect NSCLC cells in the presence of 10 μg/ml polybrene (Sigma, St Louis, MO, USA). To eliminate the O-GlcNAcylation of PGK1, pcDNA3.1( +)-PGK1 wild-type or mutant (T255V) plasmids were obtained from Genewiz (Suzhou, China). The transfections of plasmids were performed using the jetPRIME reagent. To upregulate miR-24-3p, RNAi-mate reagent was used to transfect cells with chemically synthesized miRNA mimics (GenePharma, Shanghai, China). All shRNA sequences are shown in (Additional file 3: Table S1).

## Quantitative real-time PCR (qRT-PCR) and Western blotting

The RNA extraction process utilized an RNA extraction kit from Tiangen(Beijing, China). Reverse transcription was executed with the HiFiScript cDNA Synthesis Kit from CWBIO(Beijing, China). The SYBR® Premix EX Taq II kit from TaKaRa (Dalian, China) was employed for the PCR reactions. The primer sequences are presented in (Additional file 4: Table S2). Protein extraction was performed utilizing a commercial kit (Proteintech, Wuhan, China). Equal quantities of protein (10-20 μg) were separated on an 8–15% SDS-PAGE and transferred onto nitrocellulose membranes (Merck Millipore, Billerica, MA, USA). The antibodies were acquired from the sources listed below: PGK1(17,811–1-AP, Proteintech), OGT(11,576–2-AP, Proteintech), MCM4(13,043–1-AP, Proteintech), O-GlcNAc (MA1-072, Thermo Fisher Scientific, Waltham, MA, USA), ERK1 + ERK2 (ab184699, Abcam, Shanghai, China), p-ERK1 (T202) + p-ERK2(T185) (ab214036, Abcam), and GAPDH (60,004–1-lg, Proteintech). The visualization of proteins was achieved through the utilization of enhanced chemiluminescence (Proteintech).

## Detection of cell proliferation, migration, and invasion

Cell proliferation was measured using the Cell Counting Kit-8 (CCK-8, Beyotime, Shanghai, China) and colony formation assays, while the Transwell chambers (Corning Incorporated, Corning, NY, USA) were employed to monitor cell migratory and invasive capabilities. These procedures were conducted in accordance with previously established protocols [15].

## Flow cytometry

The cell cycle and apoptosis protocols were executed in accordance with the instructions outlined in the cell cycle analysis kit (C1052, Beyotime) and Annexin V-PE/7-AAD apoptosis kit (C1062M, Beyotime). Data were collected using a flow cytometer (BD Biosciences, San Jose, USA).

## Xenograft model

The Institutional Animal Care and Use Committee of the Hubei University of Medicine approved the animal experiments. BALB/c nude mice (3–4 weeks) were procured from the Experimental Animal Centre of the Hubei University of Medicine, and 2 × 10^6^ cells were subcutaneously inoculated into the right flank of mice. Tumor size was measured weekly for a period of four weeks. Tumor tissues were subjected to staining with Ki-67 (27,309–1-AP, Proteintech) or PGK1, following the methodology previously described by our research team [15].

## Co-immunoprecipitation (Co-IP)

Co-IP was done using Classic IP/Co-IP kit (Pierce) according to the manufacturer's protocol(Thermo Fisher Scientific). Briefly, cells with indicated treatment were lysed in IP lysis buffer, and the resulting supernatants were subjected to incubation with the appropriate antibodies on a rotating wheel. Protein A/G-agarose beads were introduced to precipitate the complexes. The eluted proteins were separated through SDS-PAGE and subjected to analysis via Western blotting.

## Luciferase assay

The 3'-UTR of OGT mRNA, either in its wild-type or mutant form, was amplified through PCR and subsequently subcloned into the pGL3 vector (GenePharma). Luciferase activity was quantified as previously reported [16].

## RNA-seq

Novogene (Tianjin, China) conducted RNA isolation, quality control, library preparation, and high-throughput sequencing using an Illumina HiSeq system (Illumina, San Diego, CA, USA). Data processing was performed according to our previous protocol [17]. Log2(fold change) > 1 and FDR < 0.05 were established as the cut-offs for identifying differentially expressed genes (DEGs). The clusterProfiler R package was utilized to perform Gene Ontology (GO) annotation and Kyoto Encyclopedia of Genes and Genomes (KEGG) pathway enrichment of DEGs [18].

## Statistical analysis

The data were presented as means ± SD and subjected to analysis using various methods, including Student’s t-test, Mann–Whitney U test, ANOVA, Pearson correlation analysis, and Log-Rank test. Significance was set at *P* < 0.05.

## Results

### PGK1 is overexpressed in NSCLC

**Fig. 1:**
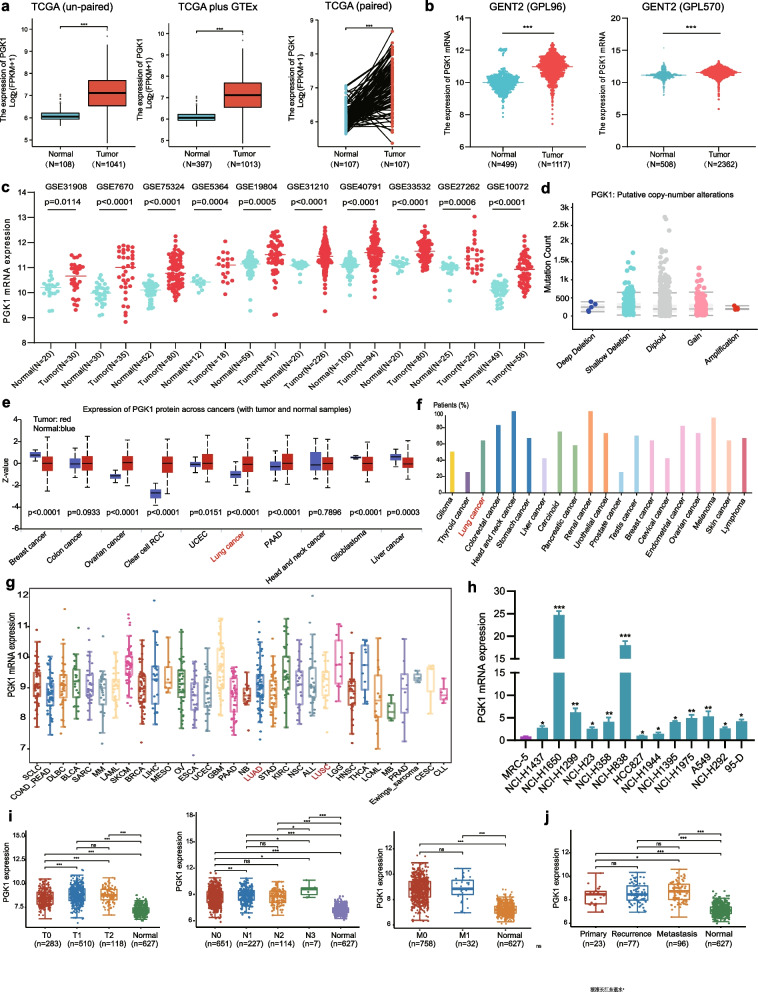
Expression analysis of PGK1 in NSCLC tissues and cell lines. **a** Comparison of PGK1 mRNA expression in NSCLC samples and normal samples using the TCGA and GTEx databases. **b** Differential mRNA expression of PGK1 between NSCLC tissues and normal tissues based on the GENT2 database. **c** Assessment of PGK1 mRNA levels in tumors and normal tissues of NSCLC patients from the GEO database. **d** PGK1 mutations and copy number alterations in NSCLC tissues examined by the cBioPortal database. **e** The protein levels of PGK1 in various normal and tumor tissues from the CPTAC database. **f** PGK1 protein expression across different tumor tissues in the HPA database. **g** The mRNA expression of PGK1 in tumor cell lines from the CCLE database. **h** Identification of PGK1 mRNA expression in NSCLC cells using qRT-PCR. (i) The association between PGK1 mRNA expression and tumor stage by analyzing the TCGA dataset. (j) The mRNA levels of PGK1 in primary and metastatic NSCLC specimens from the TCGA cohort. ^*^P < 0.05, ^**^P < 0.01, ^***^P < 0.001, and ns, not significant

The analysis of RNA-seq data obtained from the TCGA, GTEx, GENT2, and GEO databases revealed a frequent upregulation of PGK1 mRNA in both non-paired and paired NSCLC tissues (Fig. 1a-c). Genetic alteration analysis of the NSCLC cohort using the cBioportal tool demonstrated the presence of PGK1 gene copy-number gain or amplification (Fig. 1d). Additional investigation utilizing the HPA and CPTAC programs revealed a notable overexpression of PGK1 protein in most malignant tissues, including NSCLC (Fig. 1e, f). Moreover, the examination of the CCLE database indicated that NSCLC cell lines exhibited high levels of PGK1 mRNA (Fig. 1g). Confirmation of heightened PGK1 mRNA expression in NSCLC cells was achieved via qRT-PCR analysis (Fig. 1h). Subsequently, data from TCGA were extracted to investigate whether PGK1 expression was correlated with clinicopathological characteristics of NSCLC patients. Notably, significant associations were observed between PGK1 expression and gender, T-stage, and N-stage (Additional file 5: Table S3). PGK1 mRNA expression also displayed an increasing trend with the advancement of N-stage and T-stage (Fig. 1i). Furthermore, metastatic tumors exhibited higher levels of PGK1 mRNA compared to primary tumors (Fig. 1j). It is evident that PGK1 is important in NSCLC based on these observations.

## PGK1 overexpression in NSCLC predicts poor prognosis

**Fig. 2:**
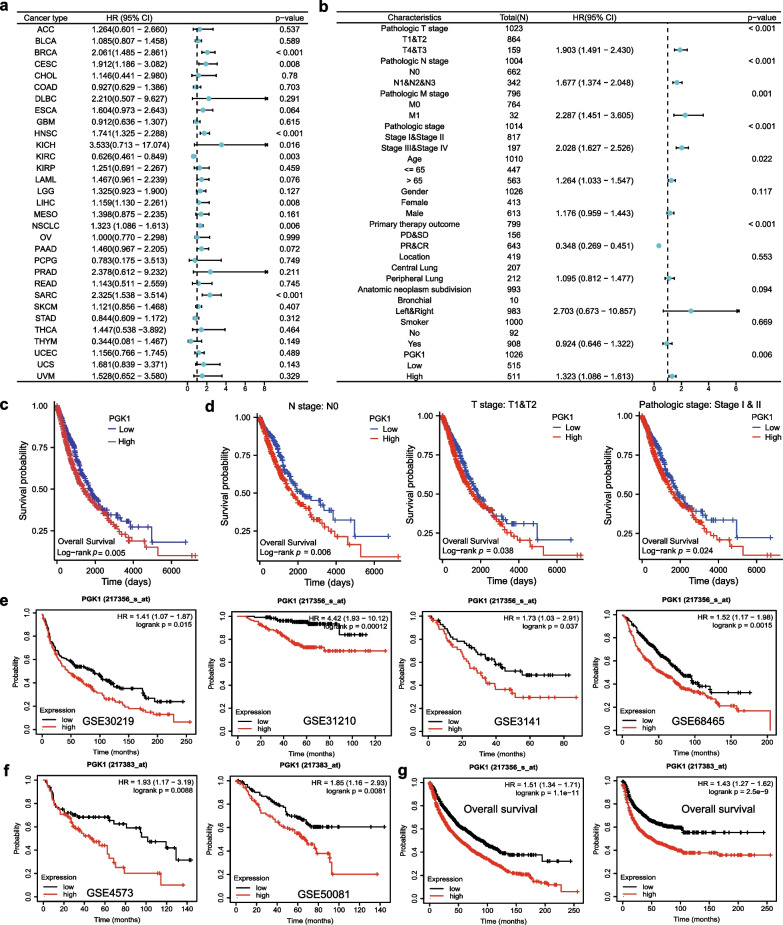
Survival analysis of NSCLC patients based on PGK1 expression. **a** Univariate Cox regression analyses in various TCGA cohorts. **b** Forest plots derived from univariate Cox analysis of the TCGA-NSCLC cohort. **c** Kaplan–Meier curves for all NSCLC patients from the TCGA database. **d** Kaplan–Meier curves for different TCGA-NSCLC subgroups. **e** Kaplan–Meier analysis using the GEO datasets with the probe id 217356_s_at. **f** Kaplan–Meier analysis using the GEO datasets with the probe id 217383_at. **g** The prognostic value of PGK1 in the Kaplan–Meier plotter database

The survival information was downloaded from the TCGA platform and subjected to univariate Cox regression analysis. We discovered that PGK1 was a significant risk factor for overall survival across diverse cancer types, including NSCLC (Fig. 2a). Additional risk factors related to the overall survival of NSCLC patients were presented in Fig. 2b. According to the multivariate Cox regression analysis, PGK1 did not attain statistical significance (Additional file 6: Table S4). Based on the Log-Rank test, a notable correlation was found between increased PGK1 expression and reduced overall survival (Fig. 2c). Subsequently, the prognostic significance of PGK1 was evaluated in different subcategories of NSCLC patients. We observed that patients with low PGK1 levels exhibited superior overall survival compared to those with high PGK1 levels in the following subgroups: N0, T1-T2, and stage I + II (Fig. 2d). Similarly, the analyses based on the GEO and Kaplan–Meier plotter databases revealed a significant inverse relationship between PGK1 expression and overall survival (Fig. 2e-g). These findings indicate that PGK1 may serve as a prognostic biomarker for NSCLC.

## PGK1 promotes NSCLC growth and metastasis both in vitro and in vivo

**Fig. 3:**
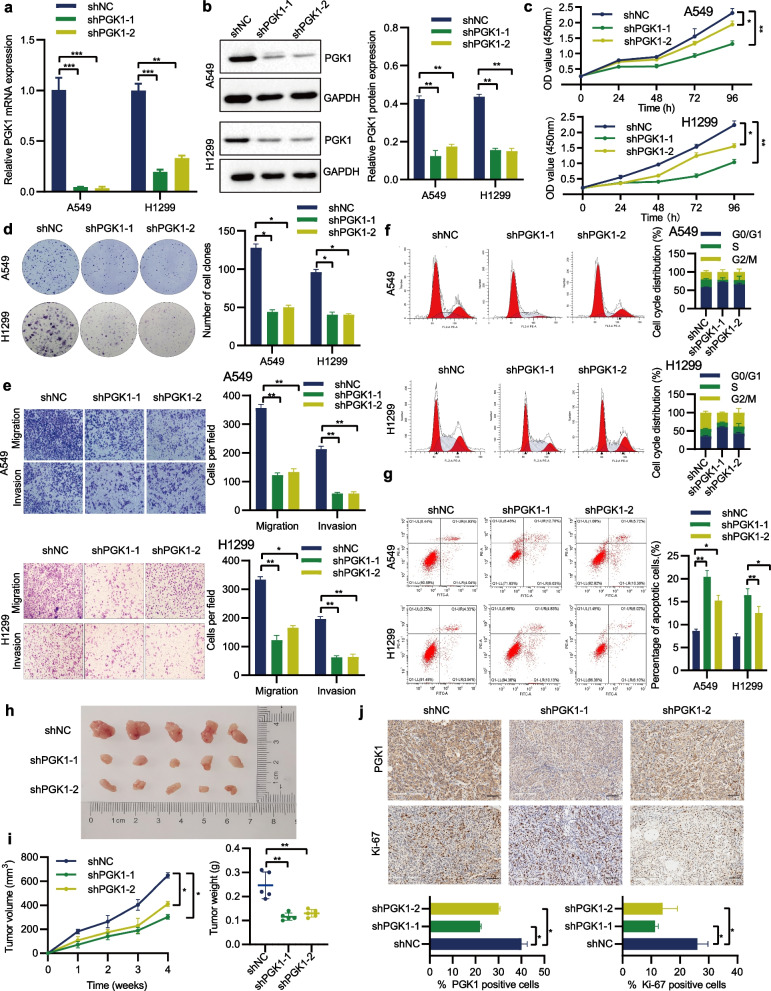
Knockdown of PGK1 suppresses NSCLC growth and metastasis. **a** The expression of PGK1 mRNA was detected using qRT-PCR. **b** The expression of PGK1 protein was analyzed through Western blotting. **c** Cell proliferation was assessed via the CCK-8 assay. **d** The Colony formation assay was utilized to evaluate the cell colony-forming ability. **e** The Transwell assay was employed to measure cell migration and invasion. **f** Flow cytometry was conducted to examine cell cycle distribution. **g** Flow cytometry was performed to monitor cell apoptosis. **h** Representative images of xenograft tumors in nude mice. **i** Assessment of the size and weight of xenograft tumors in nude mice. **j** Immunohistochemical examination of PGK1 and Ki-67 expression in tumors dissected from nude mice. ^*^P < 0.05, ^**^P < 0.01, and ^***^P < 0.001

**Fig. 4:**
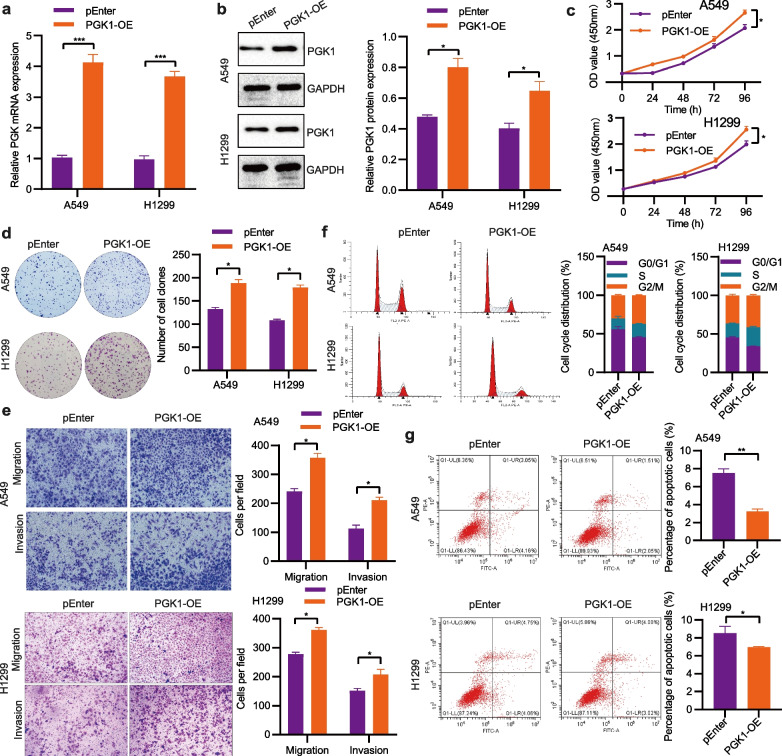
Overexpression of PGK1 enhances NSCLC growth and metastasis. **a** The quantification of PGK1 mRNA was accomplished using qRT-PCR. **b** The analysis of PGK1 protein expression was conducted via Western blotting. **c** The evaluation of cell proliferation was performed utilizing the CCK-8 assay. **d** The assessment of cell colony-forming ability was executed through the utilization of the colony formation assay. **e** The measurement of cell migration and invasion was carried out using the Transwell assay. **f** The examination of cell cycle distribution was conducted through flow cytometry. **g** The monitoring of cell apoptosis was performed via flow cytometry. ^*^P < 0.05, ^**^P < 0.01, and ^***^P < 0.001

To comprehensively describe the biological function of PGK1 in NSCLC, we selected two NSCLC cell lines (A549 and H1299) that exhibited a moderate expression level of PGK1 for the follow-up studies (as depicted in Fig. 1h). Our initial approach was to deplete endogenous PGK1 expression by shRNA (Fig. 3a, b). The CCK-8, colony formation, and Transwell assays showed that the depletion of PGK1 inhibited the proliferative, colony-forming, migratory, and invasive abilities in both cell lines (Fig. 3c-e). By flow cytometry analysis, it was found that PGK1 depletion caused cell cycle arrest in G0/G1 phase as well as cell apoptosis (Fig. 3f-g). Subsequently, an animal experiment was conducted to validate the impact of PGK1 on tumorigenesis, wherein tumor growth curves, tumor weights, and Ki-67 (proliferation biomarker) levels were documented. As anticipated, a noteworthy suppression of tumor growth was observed upon the depletion of PGK1 (Fig. 3h-j). The second methodology involved upregulating PGK1 expression through the utilization of overexpression plasmids (Fig. 4a, b). The subsequent functional experiments provided evidence that the enforced expression of PGK1 in A549 and H1299 cells considerably augmented cell proliferation, colony formation, migration, invasion, cell-cycle progression, and decreased cell apoptosis (Fig. 4c-g). Thus, PGK1 overexpression is the main factor responsible for NSCLC progression.

## OGT-mediated O-GlcNAcylation is essential for PGK1-driven NSCLC progression

**Fig. 5:**
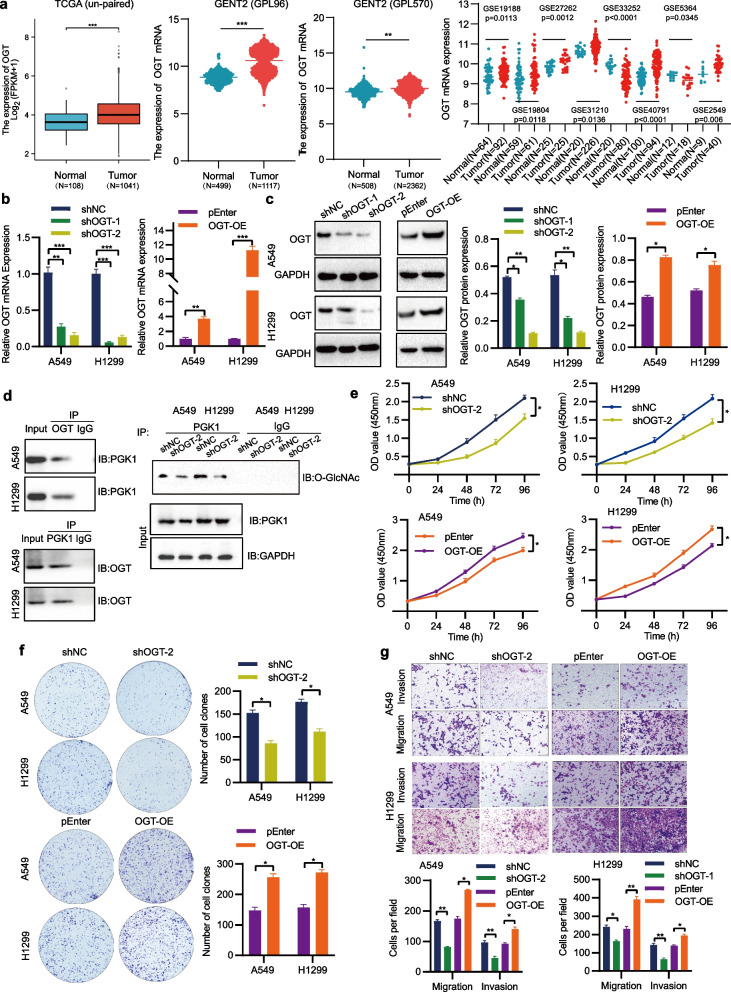
PGK1 interacts with and is O-GlcNAcylated by OGT. **a** The evaluation of OGT mRNA levels in both tumor and normal tissues of NSCLC patients from the TCGA, GENT2, and GEO databases. **b** The detection of OGT mRNA expression was performed using qRT-PCR. **c** The analysis of OGT protein expression was conducted through Western blotting. **d** Co-IP was employed to investigate the interaction between PGK1 and OGT. **e** The assessment of cell proliferation was accomplished by means of the CCK-8 assay. **f** The monitoring of cell colony-forming ability was executed using the colony formation assay. **g** The measurement of cell migration and invasion was carried out utilizing the Transwell assay. ^*^P < 0.05, ^**^P < 0.01, and ^***^P < 0.001

**Fig. 6:**
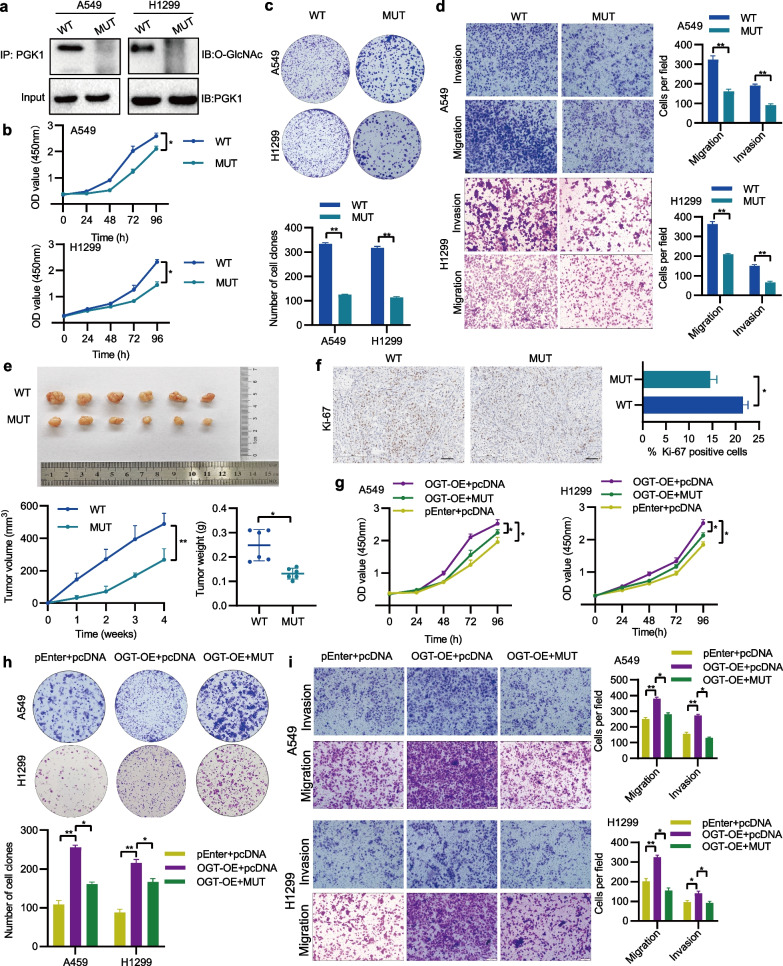
O-GlcNAcylation regulates PGK1 function. **a** The detection of O-GlcNAcylation levels was performed through immunoprecipitation. **b**-**d** The impact of PGK1 mutation on NSLCL cell growth, migration, and invasion was assessed using the CCK-8, colony formation, and Transwell assays, respectively. **e** An animal experiment was conducted to evaluate the effect of PGK1 mutation on tumor growth. **f** Immunohistochemical staining of Ki-67 in xenograft tumors from the nude mice. **g**-**i** Cell growth, migration, and invasion in different groups were separately measured by the CCK-8, colony formation, and Transwell assays. MUT, pcDNA3.1( +)-PGK1 wild-type plasmid; MUT, pcDNA3.1( +)- PGK1 mutant plasmid. ^*^P < 0.05,^**^P < 0.01

OGT catalyzes the process of O-GlcNAcylation. To explore the influence of OGT on PGK1, an initial assessment of OGT expression in NSCLC tissues was conducted, followed by modulation of its expression in NSCLC cells. Data from the TCGA, GENT2, and GEO databases demonstrated that NSCLC tissues expressed higher levels of OGT mRNA (Fig. 5a). After integrating the analysis of GEO and TCGA data, we found that OGT expression was also negatively associated with overall survival (Additional file 1: Figure S1). Based on the results of qRT-PCR and Western blotting analyses, the modulation of PGK1 expression in A549 and H1299 cells was achieved through the utilization of shRNA or pENTER-OGT (Fig. 5b, c). Co-IP assays showed that OGT interacted with PGK1 and affected its O-GlcNAcylation (Fig. 5d). A reduction in PGK1 O-GlcNAcylation occurred when OGT was suppressed. Notably, the manipulation of OGT did not alter PGK1 protein expression. By performing in vitro functional assays, it was determined that OGT played a promotive role in the growth and metastasis of NSCLC cells (Fig. 5e-g). To address whether O-GlcNAcylation directly regulates the function of PGK1, a T255 mutant was generated to abolish the putative O-GlcNAc site on PGK1. The results indicated that the T255 mutant effectively abrogated PGK1 O-GlcNAcylation (Fig. 6a). Meanwhile, in vitro and in vivo experiments displayed that the T255 mutant reduced NSCLC cell growth and metastasis (Fig. 6b-f). Moreover, the mutation of the PGK1 O-GlcNAcylation site exhibited the potential to mitigate the stimulatory effects of OGT overexpression on cellular growth and metastasis (Fig. 6g-i). The above observations demonstrate that OGT-mediated PGK1 O-GlcNAcylation is a key player in the malignant progression of NSCLC.

## OGT expression is negatively controlled by miR-24-3p

**Fig. 7:**
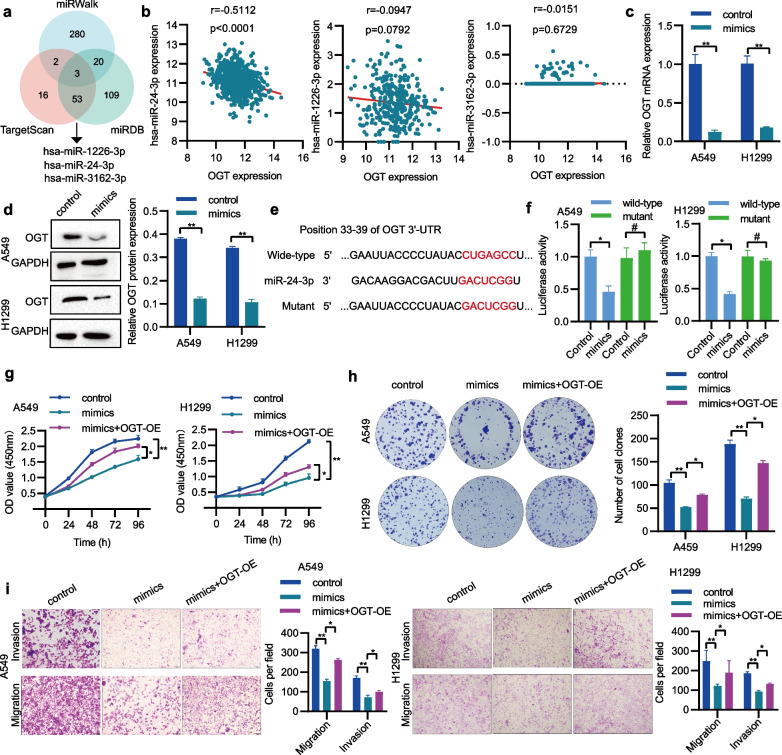
miR-24-3p binds to and negatively modulates OGT expression. **a** Venn diagram depicting the intersection of predicted miRNAs targeting OGT. **b** The relationship between OGT and miRNAs in NSCLC patients was examined via LinkedOmics analysis. **c** qRT-PCR was employed to detect OGT mRNA expression. **d** Western blotting was utilized to analyze OGT protein expression. **e** TargetScan was used to predict miR-24-3p binding sites in the 3′-UTR of OGT mRNA. **f** Direct interaction between miR-24-3p and OGT was confirmed by the dual-luciferase reporter assay. **g** The assessment of cell proliferation was accomplished via the CCK-8 assay. **h** The monitoring of cell colony-forming ability was executed using the colony formation assay. **i** The measurement of cell migration and invasion was carried out utilizing the Transwell assay. ^*^P < 0.05, ^**^P < 0.01, and ^#^*P* > 0.05

To uncover the cause of the elevated OGT expression in NSCLC, our analysis focused on miRNAs due to their crucial role in regulating gene transcription. By utilizing a range of analytical tools such as TargetScan, miRDB, and miRWalk, we identified three miRNAs that exhibited potential binding affinity with OGT (Fig. 7a). Correlation analysis using the LinkedOmics database revealed that only miR-24-3p had a significant negative correlation with OGT in NSCLC (Fig. 7b). To assess whether OGT expression was modulated by miR-24-3p, NSCLC cells were treated with miR-24-3p mimics. Through qRT-PCR and Western blotting, it was discovered that miR-24-3p mimics inhibited the mRNA and protein expression of OGT (Fig. 7c, d). According to TargetScan, OGT contains a conserved binding site for miR-24-3p in its 3′-UTR (Fig. 7e). The confirmation of the direct binding between miR-24-3p and OGT 3′-UTR was achieved through the utilization of a dual-luciferase reporter assay (Fig. 7f). To figure out whether miR-24-3p/OGT axis was involved in NSCLC progression, a series of functional assays were conducted. We observed that the introduction of miR-24-3p resulted in a reduction in cell growth and metastasis. However, these effects were reversed upon the overexpression of OGT (Fig. 7g-i). Hence, miR-24-3p acts as a negative regulator of OGT in NSCLC.

## PGK1 activates the MAPK/ERK pathway

**Fig. 8:**
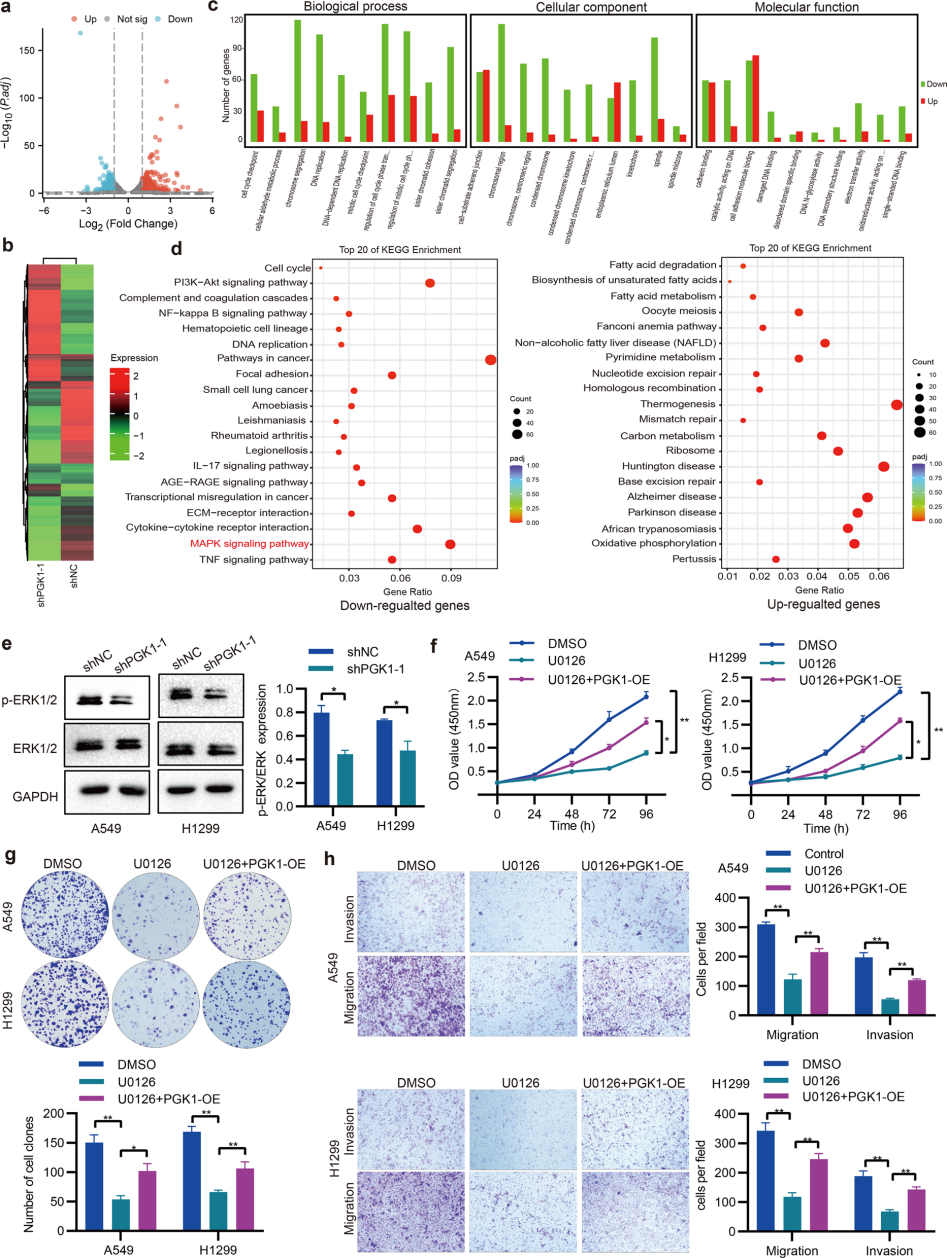
MAPK/ERK pathway is activated by PGK1. **a** The RNA-seq data was visualized using Volcano plots. **b** The Heatmap was generated with the RNA-seq data. **c** GO analysis of DEGs. **d** KEGG enrichment analysis of DEGs. **e** The ERK and p-ERK expression levels were detected through the utilization of Western blotting. **f** The CCK-8 assay was employed to accomplish the assessment of cell proliferation. **g** The execution of monitoring cell colony-forming ability was achieved by utilizing the colony formation assay. **h** The Transwell assay was utilized for the measurement of cell migration and invasion. ^*^P < 0.05,^**^P < 0.01

To investigate how PGK facilitates NSCLC progression, we conducted RNA-seq experiments utilizing A549 cells with and without PGK1-knockdown. We observed that the knockdown of PGK1 increased 1774 genes and decreased 2065 genes (Fig. 8a, b, Additional file 7: Table S5). DEGs were analyzed using GO and KEGG (Fig. 8c, d). Notably, the downregulated genes were predominantly enriched in the MAPK pathway. Considering that ERK is a key component of the MAPK pathway, the protein expression of p-ERK and ERK was examined via Western blotting (Fig. 8e). By knocking down PGK1, the activity of ERK was reduced (measured as p-ERK to ERK ratio). Subsequently, U0126 (an inhibitor of MAPK/ERK) was added to determine if PGK1-mediated malignant phenotypes were associated with the MAPK/ERK pathway. Our findings demonstrated that U0126 exerted suppressive effects on cell growth and metastasis, which were, however, counteracted by the overexpression of PGK1 (Fig. 8f-h). These data imply that PGK1 triggers the activation of MAPK/ERK in NSCLC.

## PGK1 upregulates the expression of MCM4

**Fig. 9:**
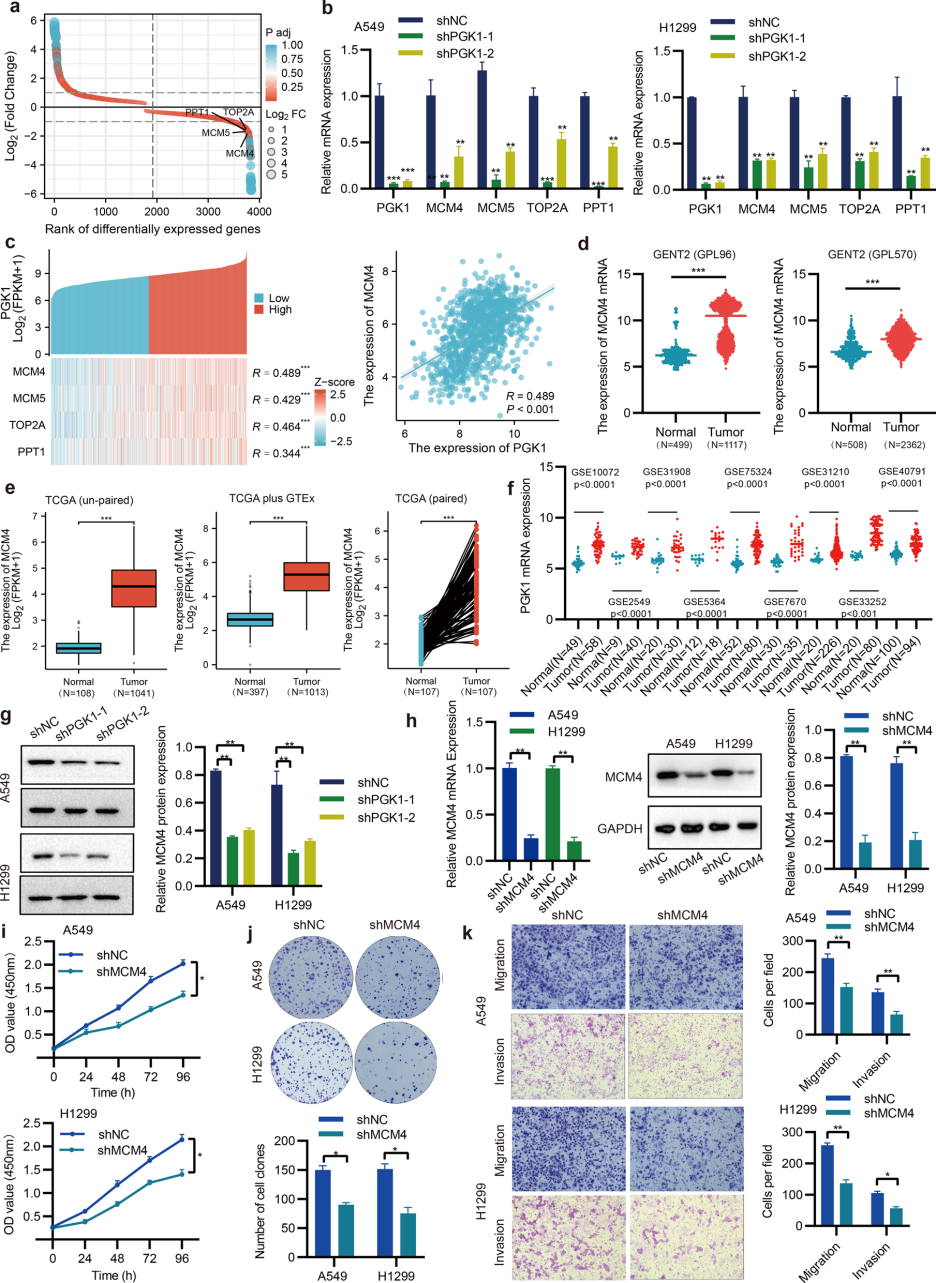
MCM4 expression is upregulated by PGK1. **a** The ranking of differential gene expression was performed using RNA-seq data. **b** The detection of mRNA expression for specific genes was accomplished through qRT-PCR. **c** The examination of the correlation between PGK1 and chosen genes was conducted by utilizing the TCGA database. **d**-**f** The assessment of MCM4 mRNA expression in NSCLC tissues was carried out via the utilization of TCGA, GENT2, and GEO databases. **g** The Western blotting technique was employed to validate the protein expression of MCM4. **h** Analysis of MCM4 expression by qRT-PCR and Western blotting. **i**-**k** The CCK-8, colony formation, and Transwell assays were utilized to independently measure cell growth, migration, and invasion across different groups. ^*^P < 0.05, ^**^P < 0.01, and ^***^P < 0.001

**Fig. 10:**
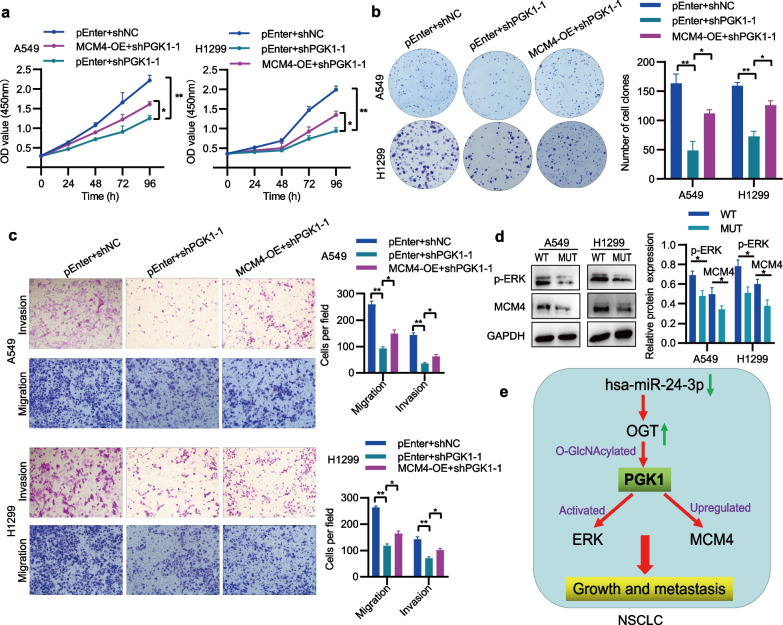
Role of MCM4 in PGK1-mediated NSCLC progression. **a** Cell proliferation was detected via the CCK-8 assay. **b** Cell colony-forming ability was measured using the colony formation assay. **c** Cell migration and invasion were examined utilizing the Transwell assay. **d** The effects of PGK1 mutation on p-ERK and MCM4 levels were detected through Western blotting. **e** Diagram showing the regulation mechanism of PGK1 in NSCLC progression. MUT, pcDNA3.1( +)-PGK1 wild-type plasmid; MUT, pcDNA3.1( +)- PGK1 mutant plasmid. ^*^P < 0.05,^**^P < 0.01

To ascertain the key genes regulated by PGK1, we conducted qRT-PCR on the same RNA samples to verify the sequencing data. MCM4, MCM5, TOP2A, and PPT1 were selected based on their relative abundance, fold change, and adjust p-value (Fig. 9a). In line with our expectations, the knockdown of PGK1 notably reduced mRNA levels of MCM4, MCM5, TOP2A, and PPT1 (Fig. 9b). Among these genes, MCM4 exhibited the strongest positive correlation with PGK1 in NSCLC from the TCGA database (Fig. 9c). The data obtained from the TCGA, GENT2, and GEO databases confirmed that MCM4 was highly expressed in NSCLC tissues (Fig. 9d-f). Meanwhile, the results of survival analysis from GEO and TCGA indicated that high expression of MCM4 was associated with shorter overall survival (Additional file 2: Figure S2). Thus, MCM4 was chosen for further study. To validate the modulation of MCM4 by PGK1, Western blotting analysis was conducted, revealing that MCM4 protein expression was suppressed in A549 and H1299 cells with PGK1 knockdown (Fig. 9g). To explore whether MCM4 was involved in PGK1-mediated NSCLC progression, MCM4 expression was silenced by shRNA (Fig. 9h). The functional experiments demonstrated that MCM4 silencing repressed cell proliferation, colony formation, migration, and invasion (Fig. 9i-k). Additionally, the restoration of MCM4 expression abolished the inhibited proliferation, colony formation, and invasion of NSCLC cells that were caused by PGK1 knockdown (Fig. 10a-c). It was noteworthy that the mutation of the PGK1 O-GlcNAcylation site could inhibit p-ERK and MCM4 levels in A549 and H1299 cells (Fig. 10d). Therefore, MCM4 is a crucial downstream target of PGK1 in NSCLC.

## Discussion

Many tumors are known to be affected by PGK1. Nevertheless, the precise role of PGK1 in NSCLC remains elusive. This study has confirmed the distinct upregulation of PGK1 in NSCLC, which is a major contributor to poor prognosis. Functionally, elevated expression of PGK1 is necessary for NSCLC growth and metastasis. Mechanistically, the miR-24-3p/OGT axis governs O-GlcNAcylation, which is indispensable for PGK1-mediated NSCLC progression. Moreover, both ERK and MCM4 have been identified as downstream targets of PGK1 (Fig. 10e). To our knowledge, this is the first comprehensive report to integrate multiple data from bioinformatics analyses, cellular experiments, and nude mouse xenograft models, to examine the oncogenic role of PGK1 in NSCLC.

NSCLC is a disease with high mortality and morbidity, and altered glucose metabolism has been identified as a distinguishing feature. Extensive documentation has shown enhanced glucose absorption and heightened glycolytic pathway activity in NSCLC [19, 20]. Consequently, there is a growing emphasis on glycometabolism-related enzymes. Although the canonical activities of these enzymes are typically linked to the regulation of carbohydrate and energy metabolism, certain enzymes have been discovered to possess non-canonical or non-metabolic functions [21]. The dysregulated expression of metabolic enzymes with such properties is pivotal in controlling gene transcription, cell survival, DNA damage repair, cell-cycle progression, and apoptosis [22–24]. Thus, comprehending the non-classical roles of glycometabolism-related enzymes can offer valuable biomarkers for tumor prognosis and treatment. According to a previous report, PGK1 serves not only as a metabolic enzyme but also as an oncogene, contributing to tumorigenesis and progression [4]. In this study, we primarily investigated the non-metabolic function of PGK1 in NSCLC. Our findings from multiple assays, including CCK-8, colony formation, Transwell, and flow cytometry, revealed that the knockdown of PGK1 inhibited NSCLC cell proliferation, colony formation, migration, and invasion, while inducing G0/G1 cell cycle arrest and apoptosis. Additionally, tumorigenicity experiments displayed that PGK1 knockdown suppressed tumor formation in nude mice. Our results provide insight into the mechanisms underlying NSCLC progression and highlight PGK1 as a promising therapeutic target for NSCLC.

PGK1 undergoes diverse post-translational modifications that contribute to its multifaceted functions. For example, acetylation of PGK1 initiates hypoxia-induced autophagy and sustains tumor growth [25]. Phosphorylation of PGK1 enhances glycolysis by altering substrate affinity, thereby promoting tumorigenesis [26]. When PGK1 is ubiquitinated, the oncogenic AKT/mTOR pathway is inactivated [27]. PGK1 succinylation is capable of influencing epileptic seizures [28]. O-GlcNAcylation at T255 activates PGK1 and mediates its mitochondrial translocation [8]. Notably, O-GlcNAcylation has gradually become a research hotspot in recent years. Numerous diseases, including cardiovascular ailments, diabetes, and cancers, have been linked to aberrant O-GlcNAcylation [13, 29]. In NSCLC, we identified PGK1 as a protein that interacted with OGT and was O-GlcNAcylated. The inhibition of PGK1 O-GlcNAcylation through OGT silencing or T255 mutation could weaken PGK1-induced malignant phenotypes in NSCLC cells. Based on the confirmation of the importance of OGT-mediated O-GlcNAcylation in the non-metabolic function of PGK, our work delves further into the factors that modulate OGT expression. It is widely acknowledged that gene expression is subject to regulation by various mechanisms, such as non-coding RNAs, alternative splicing, DNA methylation, histone acetylation, and transcriptional initiation. Previous studies have suggested that certain miRNAs can regulate OGT expression [30, 31]. According to our findings, decreased miR-24-3p acted as a pivotal modulator in maintaining OGT abundance. Thus, we have reason to believe that a low miR-24-3p level leads to an increase in OGT expression, thereby facilitating the O-GlcNAcylation of PGK1, ultimately resulting in the manifestation of its non-metabolic function. However, the correlation between O-GlcNAcylation and the non-canonical function of PGK1 warrants thorough investigation.

PGK1 exhibits oncogenic properties by affecting multiple signaling pathways and effector molecules. For instance, the relationship between PGK1 and CXCR4/CXCL12/β-catenin has been established in gastric cancer and hepatocellular carcinoma [32, 33]. PGK1 has been linked to AKT phosphorylation in oral squamous cell carcinoma [27]. In liver cancer, PGK1 suppresses cell death through modulation of PRAS40, while in colon cancer, it upregulates the expression of EGR1, a metastasis-related factor [9, 34]. In this investigation, we found that the malignant progression of NSCLC was facilitated by PGK1 through the activation of ERK. The findings are consistent with a prior study showing PGK1 positively regulates ERK [5]. MCM4, an essential member of the minichromosomal maintenance protein family, has been shown to play a crucial role in numerous human cancers, particularly in NSCLC [35, 36]. Here, we demonstrated that high levels of MCM4 were positively related to the malignant phenotypes of NSCLC cells. Meanwhile, PGK1 modulated MCM4 expression, leading to its upregulation in NSCLC. Our study uncovers novel regulatory mechanisms underlying ERK pathway activation and MCM4 upregulation in NSLCL. However, further investigation is required to fully elucidate the intricate interplay between PGK1, ERK, and MCM4.

## Conclusions

Our findings suggest that PGK1 acts as an oncogene in NSCLC, with its function dependent on the miR-24-3p/OGT axis mediated-O-GlcNAcylation. PGK1 promotes NSCLC progression by activating ERK and upregulating MCM4. In-depth research on PGK1 may offer a novel and promising treatment for NSCLC.

### Supplementary Information

**Additional file1**: Kaplan-Meier curves for all NSCLC patients based on OGT expression from the TCGA and GEO databases.

**Additional file2**: Kaplan-Meier curves for all NSCLC patients based on MCM4 expression from the TCGA and GEO databases.

**Additional file3**: Sequences of shRNAs

**Additional file4**: Primer sequences for qRT-PCR

**Additional file5**: Associations between PGK1 expression and clinicopathological parameters in NSCLC

**Additional file6**: Multivariate Cox regression analyses of overall survival in patients with NSCLC

**Additional file7**: Differentially expressed genes identified by RNA-seq

## Data Availability

All data generated or analyzed during this study are included in this published article.

## Abbreviations

PGK1: Phosphoglycerate kinase 1
NSCLC: Non-small cell lung cancer
TCGA: The Cancer Genome Atlas
GEO: Gene Expression Omnibus
GENT2: Gene Expression Database of Normal and Tumor tissues 2
GTEx: Genotype-Tissue Expression
CELE: Cancer Cell Line Encyclopedia
HPA: Human Protein Atlas
CPTAC: Clinical Proteomic Tumor Analysis Consortium
OGT: O-GlcNAc transferase
MCM4: Minichromosome maintenance complex component 4
qRT-PCR: Quantitative real-time PCR
CCK-8: Cell Counting Kit-8
Co-IP: Co-immunoprecipitation
DEGs: Differentially expressed genes
GO: Gene Ontology
KEGG: Kyoto Encyclopedia of Genes and Genomes
3′-UTR: 3′-Untranslated region
