# Supplementary figures and images for: The oncogenic role and regulatory mechanism of PGK1 in human non-small cell lung cancer
Source: Biol Direct. 2024 Jan 2;19:1. doi: 10.1186/s13062-023-00448-9 (PMC10759362; doi:10.1186/s13062-023-00448-9)

# OGT (220594\_at)

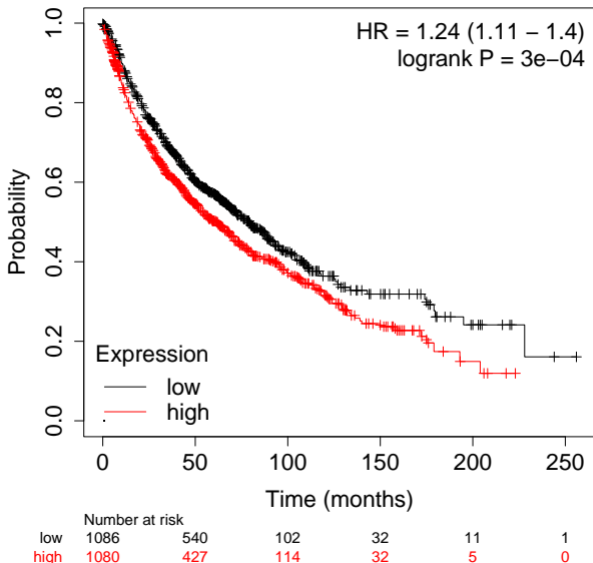

Supplement: Supplementary file 1 — Additional file1: Kaplan-Meier curves for all NSCLC patients based on OGT expression from the TCGA and GEO databases. [file 13062_2023_448_MOESM1_ESM.pdf]

# MCM4 (222037\_at)

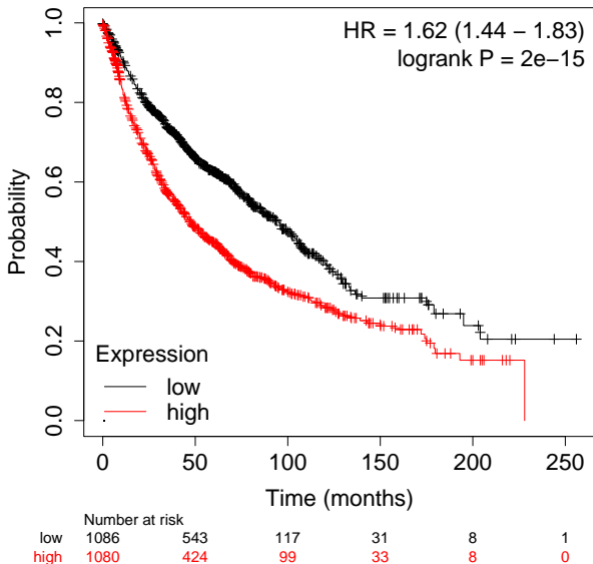

Supplement: Supplementary file 2 — Additional file2: Kaplan-Meier curves for all NSCLC patients based on MCM4 expression from the TCGA and GEO databases. [file 13062_2023_448_MOESM2_ESM.pdf]
