# Supplementary material for: The oncogenic role and regulatory mechanism of PGK1 in human non-small cell lung cancer
Source: Biol Direct. 2024 Jan 2;19:1. doi: 10.1186/s13062-023-00448-9 (PMC10759362; doi:10.1186/s13062-023-00448-9)
Supplement: Supplementary file 3 — Additional file3: Sequences of shRNAs [file 13062_2023_448_MOESM3_ESM.doc]

**Table S1.** Sequences of shRNAs

| **Name** | **Sequences (5**′**-3**′**)** |
| --- | --- |
| PGK1-shRNA1-F  PGK1-shRNA1-R  PGK1-shRNA2-F  PGK1-shRNA2-R  OGT-shRNA1-F  OGT-shRNA1-R  OGT-shRNA2-F  OGT-shRNA2-R  MCM4-shRNA-F  MCM4-shRNA-R | CCGGCTGACAAGTTTGATGAGAATGCTCGAGCATTCTCATCAAACTTGTCAGTTTTTG  AATTCAAAAACTGACAAGTTTGATGAGAATGCTCGAGCATTCTCATCAAACTTGTCAG  CCGGGCCTACTTTATGGCAGACATTCTCGAGAATGTCTGCCATAAAGTAGGCTTTTTG  AATTCAAAAAGCCTACTTTATGGCAGACATTCTCGAGAATGTCTGCCATAAAGTAGGC  CCGGTGTTGCAGATGGGTGATATATCTCGAGATATATCACCCATCTGCAACATTTTTG  AATTCAAAAATGTTGCAGATGGGTGATATATCTCGAGATATATCACCCATCTGCAACA  CCGGTTTAGCACTCTGGCAATTAAACTCGAGTTTAATTGCCAGAGTGCTAAATTTTTG  AATTCAAAAATTTAGCACTCTGGCAATTAAACTCGAGTTTAATTGCCAGAGTGCTAAA  CCGGGCGGTGCTAAAGGACTACATTCTCGAGAATGTAGTCCTTTAGCACCGCTTTTTG  AATTCAAAAAGCGGTGCTAAAGGACTACATTCTCGAGAATGTAGTCCTTTAGCACCGC |
