# Supplementary material for: The oncogenic role and regulatory mechanism of PGK1 in human non-small cell lung cancer
Source: Biol Direct. 2024 Jan 2;19:1. doi: 10.1186/s13062-023-00448-9 (PMC10759362; doi:10.1186/s13062-023-00448-9)
Supplement: Supplementary file 4 — Additional file4: Primer sequences for qRT-PCR [file 13062_2023_448_MOESM4_ESM.doc]

**Table S2.** Primer sequences for qRT-PCR

| **Name** |  | **Sequences (5′-3′)** |
| --- | --- | --- |
| PGK1 | Forward  Reverse | TGGACGTTAAAGGGAAGCGG  GCTCATAAGGACTACCGACTTGG |
| OGT | Forward  Reverse | TCCTGATTTGTACTGTGTTCGC  AAGCTACTGCAAAGTTCGGTT |
| MCM4 | Forward  Reverse | TGAACCTCTATACATGCAACGAC  CAGGGTAACGGTCAAAGAAGATT |
| GAPDH | Forward  Reverse | GACAACTTTGGCATCGTGGA  ATGCAGGGATGATGTTCTGG |
| MCM5 | Forward  Reverse | CGGCACTGGATAGAGATGCG  AGCATTCGTAGCCTGAAGTCG |
| TOP2A | Forward  Reverse | ACCATTGCAGCCTGTAAATGA  GGGCGGAGCAAAATATGTTCC |
| PPT1 | Forward  Reverse | GGAGCGGGGTATCAATGAGTC  CCACTCCGAATCTACAGGGTC |
