# Supplementary material for: The oncogenic role and regulatory mechanism of PGK1 in human non-small cell lung cancer
Source: Biol Direct. 2024 Jan 2;19:1. doi: 10.1186/s13062-023-00448-9 (PMC10759362; doi:10.1186/s13062-023-00448-9)
Supplement: Supplementary file 5 — Additional file5: Associations between PGK1 expression and clinicopathological parameters in NSCLC [file 13062_2023_448_MOESM5_ESM.doc]

**Table S3.** Associations between PGK1 expression and clinicopathological parameters in NSCLC

| Characteristics | Low expression of PGK1 | High expression of PGK1 | P value |
| --- | --- | --- | --- |
| N | 520 | 521 |  |
| Pathologic T stage, n (%) |  |  | < 0.001 |
| T1 | 179 (17.2%) | 111 (10.7%) |  |
| T2 | 259 (25%) | 327 (31.5%) |  |
| T3 | 60 (5.8%) | 60 (5.8%) |  |
| T4 | 20 (1.9%) | 22 (2.1%) |  |
| Pathologic N stage, n (%) |  |  | 0.006 |
| N0 | 351 (34.4%) | 319 (31.3%) |  |
| N1 | 100 (9.8%) | 128 (12.6%) |  |
| N2 | 53 (5.2%) | 61 (6%) |  |
| N3 | 0 (0%) | 7 (0.7%) |  |
| Pathologic M stage, n (%) |  |  | 0.785 |
| M0 | 359 (44.4%) | 418 (51.7%) |  |
| M1 | 14 (1.7%) | 18 (2.2%) |  |
| Pathologic stage, n (%) |  |  | 0.051 |
| Stage I | 292 (28.4%) | 249 (24.2%) |  |
| Stage II | 131 (12.7%) | 156 (15.2%) |  |
| Stage III | 75 (7.3%) | 93 (9%) |  |
| Stage IV | 15 (1.5%) | 18 (1.7%) |  |
| Age, n (%) |  |  | 0.136 |
| <= 65 | 236 (23.3%) | 212 (20.9%) |  |
| > 65 | 271 (26.8%) | 294 (29%) |  |
| Gender, n (%) |  |  | < 0.001 |
| Female | 242 (23.2%) | 178 (17.1%) |  |
| Male | 278 (26.7%) | 343 (32.9%) |  |
| Primary therapy outcome, n (%) |  |  | 0.098 |
| PD | 50 (6.2%) | 52 (6.4%) |  |
| SD | 37 (4.6%) | 18 (2.2%) |  |
| PR | 4 (0.5%) | 7 (0.9%) |  |
| CR | 345 (42.6%) | 297 (36.7%) |  |
| Location, n (%) |  |  | 0.903 |
| Central Lung | 99 (23%) | 111 (25.8%) |  |
| Peripheral Lung | 105 (24.4%) | 115 (26.7%) |  |
| Anatomic neoplasm subdivision, n (%) |  |  | 0.653 |
| Bronchial | 4 (0.4%) | 6 (0.6%) |  |
| Left | 205 (20.4%) | 216 (21.4%) |  |
| Right | 293 (29.1%) | 283 (28.1%) |  |
| Smoker, n (%) |  |  | 0.063 |
| No | 56 (5.5%) | 39 (3.8%) |  |
| Yes | 450 (44.3%) | 470 (46.3%) |  |
