# Supplementary material for: The oncogenic role and regulatory mechanism of PGK1 in human non-small cell lung cancer
Source: Biol Direct. 2024 Jan 2;19:1. doi: 10.1186/s13062-023-00448-9 (PMC10759362; doi:10.1186/s13062-023-00448-9)
Supplement: Supplementary file 6 — Additional file6: Multivariate Cox regression analyses of overall survival in patients with NSCLC [file 13062_2023_448_MOESM6_ESM.doc]

**Table S4.** Multivariate Cox regression analyses of overall survival in patients with NSCLC

| Characteristics | Total(N) | Hazard ratio (95% CI) | P value |
| --- | --- | --- | --- |
| Pathologic T stage | 1,023 |  | 0.003 |
| T1&T2 | 864 |  |  |
| T4&T3 | 159 | 2.444 (1.359 - 4.395) |  |
| Pathologic N stage | 1,004 |  | 0.632 |
| N0 | 662 |  |  |
| N1&N2&N3 | 342 | 1.119 (0.706 - 1.776) |  |
| Pathologic M stage | 796 |  | 0.952 |
| M0 | 764 |  |  |
| M1 | 32 | 0.968 (0.328 - 2.856) |  |
| Gender | 1,026 |  |  |
| Female | 413 |  |  |
| Male | 613 |  |  |
| Primary therapy outcome | 799 |  | < 0.001 |
| PD&SD | 156 |  |  |
| PR&CR | 643 | 0.131 (0.075 - 0.229) |  |
| Pathologic stage | 1,014 |  | 0.995 |
| Stage I&Stage II | 817 |  |  |
| Stage III&Stage IV | 197 | 0.998 (0.539 - 1.847) |  |
| Age | 1,010 |  |  |
| <= 65 | 447 |  |  |
| > 65 | 563 |  |  |
| Smoker | 1,000 |  |  |
| No | 92 |  |  |
| Yes | 908 |  |  |
| Location | 419 |  | 0.105 |
| Central Lung | 207 |  |  |
| Peripheral Lung | 212 | 1.437 (0.927 - 2.229) |  |
| Anatomic neoplasm subdivision | 993 |  |  |
| Bronchial | 10 |  |  |
| Left&Right | 983 |  |  |
| PGK1 | 1,026 |  |  |
| Low | 515 |  |  |
| High | 511 |  |  |
